# A multidisciplinary telehealth program in patients with combined chronic obstructive pulmonary disease and chronic heart failure: study protocol for a randomized controlled trial

**DOI:** 10.1186/s13063-016-1584-x

**Published:** 2016-09-22

**Authors:** Palmira Bernocchi, Simonetta Scalvini, Tiziana Galli, Mara Paneroni, Doriana Baratti, Ottavia Turla, Maria Teresa La Rovere, Maurizio Volterrani, Michele Vitacca

**Affiliations:** 1 Continuity Care Unit and Telemedicine Service, Fondazione Salvatore Maugeri IRCCS, Via G Mazzini 129, 25065 Lumezzane, BS Italy; 2 Cardiac Rehabilitation Division, Fondazione Salvatore MaugeriI RCCS, Lumezzane, Brescia Italy; 3 Respiratory Rehabilitation Division, Fondazione Salvatore Maugeri IRCCS, Lumezzane, Brescia Italy; 4 Cardiac Rehabilitation Division, Fondazione Salvatore Maugeri IRCCS, Montescano, Pavia Italy; 5 Cardiology Department San Raffaele Pisana IRCCS, Roma, Italy

**Keywords:** Chronic obstructive pulmonary disease, Chronic heart failure, Telemedicine technology, Telehealth

## Abstract

**Background:**

Chronic obstructive pulmonary disease (COPD) and chronic heart failure (CHF) frequently coexist, significantly reducing patients’ quality of life and increasing morbidity and mortality. For either single disease, a multidisciplinary disease-management approach supported by telecommunication technologies offers the best outcome in terms of prolonged survival and reduced hospital readmissions. However, no data exist in patients with combined COPD/CHF.

We planned a randomized controlled trial to investigate the feasibility and efficacy of an integrated, home-based, medical/nursing intervention plus a rehabilitation program versus conventional care in patients with coexisting COPD/CHF. The purpose of the paper is to describe the rationale and design of the trial.

**Methods/designs:**

Patients, after inpatient rehabilitation, were randomly assigned to the intervention or control group, followed for 4 months at home, then assessed at 4 and 6 months. The intervention group followed a telesurveillance (telephone contacts by nurse and remote monitoring of cardiorespiratory parameters) and home-based rehabilitation program (at least three sessions/week of mini-ergometer exercises, callisthenic exercises and twice weekly pedometer-driven walking, plus telephone contacts by a physiotherapist). Telephone follow-up served to verify compliance to therapy, maintain exercise motivation, educate for early recognition of signs/symptoms, and verify the skills acquired. At baseline and 4 and 6 months, the 6-min Walk Test, dyspnea and fatigue at rest, oxygenation (PaO_2_/FiO_2_), physical activity profile (PASE questionnaire), and QoL (Minnesota and CAT questionnaires) were assessed. During the study, serious clinical events (hospitalizations or deaths) were recorded.

**Discussion:**

Currently, no studies have assessed the impact of a telehealth program in patients with combined COPD and CHF. Our study will show whether this approach is effective in the management of such complex, frail patients who are at very high risk of exacerbations.

**Trial registration:**

Network per la prevenzione e la sanità pubblica, CCM, Ministero della Salute “Modelli innovativi di gestione integrata telegestita ospedale-territorio del malato cronico a fenotipo complesso: studio di implementazione, validazione e impatto,” registered on 14 January 2014.

ClinicalTrials.gov Identifier: NCT02269618, registered on 17 October 2014.

**Electronic supplementary material:**

The online version of this article (doi:10.1186/s13063-016-1584-x) contains supplementary material, which is available to authorized users.

## Background

The incidence and the prevalence of chronic diseases have increased steadily in recent decades and are continuously rising. Ageing is an important current issue – patients with chronic diseases, such as chronic heart failure (CHF), chronic obstructive pulmonary disease (COPD), hypertension, and diabetes, are increasing in number and living longer [1, 2]. Till now, health care has been dominated by single-disease approaches lacking coordination and integration [3]. Although, in real life, diseases often coexist in the same patient, the approach to coexistent diseases has been largely neglected. In particular, COPD and CHF frequently coexist due to common risk factors, causing a significant deterioration in these patients’ quality of life (QoL) and increasing morbidity and mortality [2, 4]. The prevalence of COPD in CHF patients ranges from 20 to 32 %, while CHF is prevalent in more than 20 % of patients with COPD [2, 4, 5]. Patients with combined COPD and CHF are often frail and are characterized by a high risk of frequent exacerbations and often the need for rehospitalization, with the related burden of cost [2]. Each disease is an independent predictor of morbidity, mortality, impaired functional status, and health service use [6, 7]. The combination of these two diseases presents many diagnostic challenges [5]. Clinical symptoms and signs require careful interpretation, in conjunction with objective evidence for each condition. Both are chronic progressive diseases with a course that fluctuates, burdened by frequent exacerbations, through a vicious circle of dyspnea, decreased activity, new exacerbations [8, 9], depression, and social isolation, ultimately leading to death [10, 11]. Muscle mass loss and skeletal muscle wasting in these patients have serious therapeutic and clinical implications [12]. Muscle atrophy contributes to muscle fatigue during exercise, obliging patients to stop exercising even if they have not yet exhausted their heart and lung capacity [12, 13].

The weight of evidence from randomized trials in these single specific diseases indicates that a multidisciplinary disease-management approach, supported by telecommunication technologies, has the best outcome in terms of prolonged survival and reduced hospital readmission rates [14, 15]. However, in patients where these two chronic diseases coexist there is no similar evidence to date.

We previously presented, in CHF [16] and COPD patients [17], a model for a multidisciplinary care approach using structured telephone support providing medical/nursing interventions coupled with biosignal telemonitoring. This model demonstrated a possible approach for chronic single-disease treatment after hospitalization, providing a way to prevent clinical deterioration and rehospitalizations through a comprehensive, long-term intervention with regular reinforcement of patient adherence, knowledge, and skills [18]. No physical rehabilitation program was included in this model.

## Methods/design

### Aims

A personalized hospital discharge program appears to be the best approach for the follow-up care of patients with combined COPD/CHF. Particularly important for patients with multiple comorbidities is routine self-management support, consisting of education to help patients to recognize symptoms early, manage their medical devices, identify barriers to adherence to therapy such as adverse effects of drugs, and check that the intensity of physical therapy is appropriate. Our study aims to compare the feasibility and efficacy of an integrated, home-based, medical/nursing intervention plus a physical rehabilitation program versus conventional care in patients with combined COPD/CHF. The purpose of this paper is to describe the rationale and design of the trial.

### Design

**Fig. 1:**
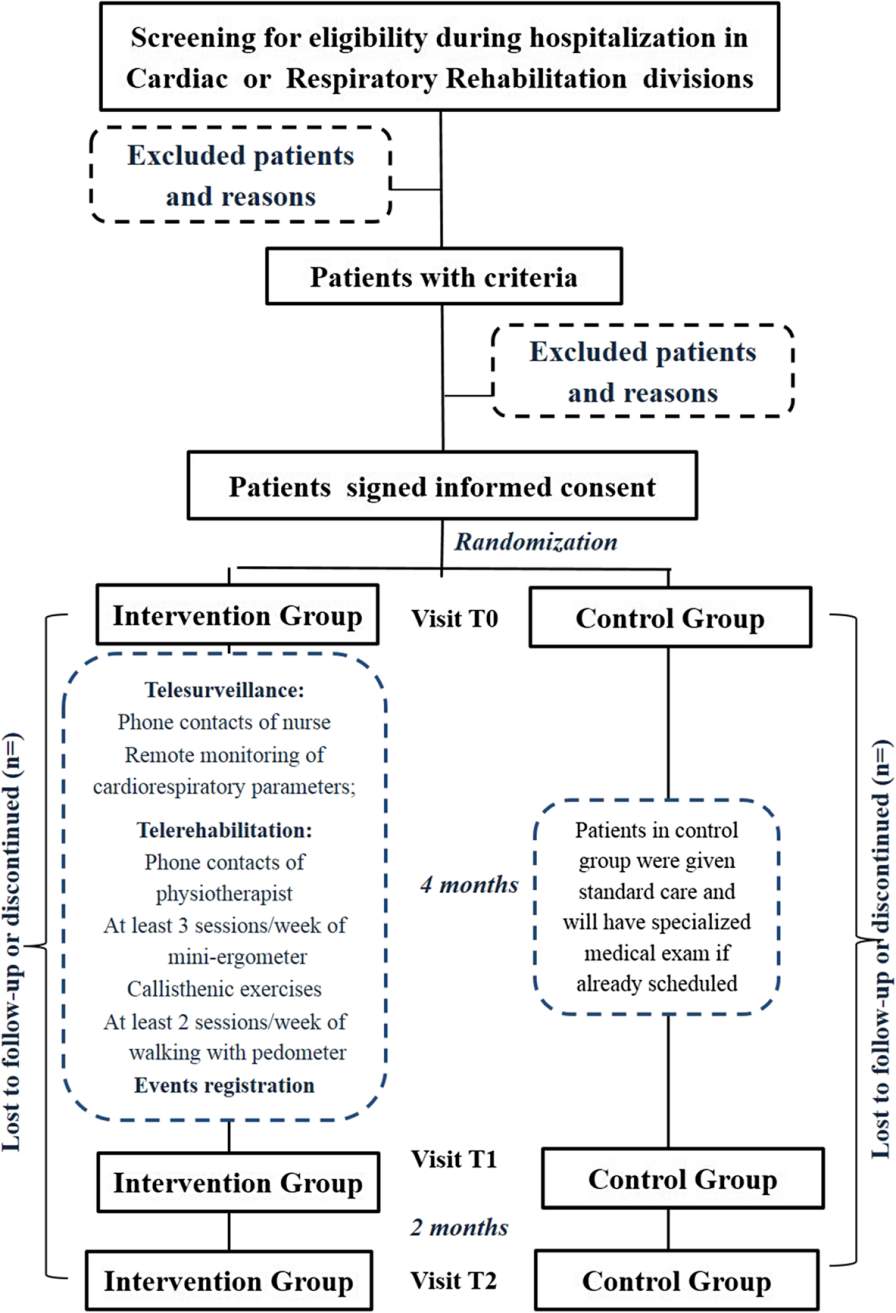
Study flow chart

This is a consecutive, multicenter, open, randomized controlled trial. All enrolled patients have been followed for 4 months at home and visited at hospital discharge (T0), after 4 months (T1) and again after another 2 months (T2), on which occasion the same tests performed at the two previous visits were repeated. A flow chart of the study design. is shown in Fig. 1.

The study strategy is registered, constructed and presented according to the recommendations for Interventional Trials (SPIRIT) [19] (SPIRIT checklist, Additional file 1) and Consolidated Standards of Reporting Trials (CONSORT) guidelines [20].

### Study sites and patient population

Patients were recruited consecutively from the Cardiology and Pulmonary Departments of three rehabilitation hospitals in Italy (Salvatore Maugeri Foundation IRCCS Institutes of Lumezzane and Montescano; and San Raffaele Pisana IRCCS, Rome). Patient selection criteria are listed in Table 1. CHF and COPD diagnoses had to be documented from at least one echocardiogram (CHF) performed within the previous 12 months under clinically stable conditions [21] and by a spirometry examination (COPD) [6] performed within the previous 12 months.

**Table 1:** Eligibility criteria

Inclusion criteria	Exclusion criteria
1. Age over 18 years	Physical activity limitations due to noncardiac and/or pulmonary conditions
2. Chronic obstructive pulmonary disease (COPD) GOLD classification (classes B, C, and D) [11]	Limited life expectancy
3. Systolic and/or diastolic heart failure (HF) New York Heart Association (NYHA) classes II, II, and IV	Severe cognitive impairments
4. At least one hospitalization or visit due to HF or COPD exacerbation in the previous 12 months	
5. Signed informed consent	

### Randomization

Consenting eligible patients were randomized to either an intervention or a control group (1:1). A computer generated tables to allocate patients in fixed blocks of 4. In order to prevent selection bias, the allocation sequence was concealed from the investigators enrolling and assessing patients, in sequentially numbered, opaque, sealed envelopes.

Due to the nature of the intervention, neither the patients nor the physicians were blinded to patients’ group allocation; however, outcome assessors and data analysts will be blinded.

### Trial structure

The trial structure of the study is described in the Table 2. A specially trained nurse-tutor (NT) and physiotherapist (PT) screened the electronic patient boards every week at each rehabilitation hospital for eligible candidates for the study and checked their eligibility in terms of the inclusion and exclusion criteria with the study doctor. If any possible candidates had been admitted in the previous week the patients were informed verbally and in writing about the study by the study team. Before discharge from hospital to home (T0), all candidates underwent assessment of arterial blood gases and nutritional status by Body Mass Index (BMI). Then patients were also administered the following evaluations or scales:

**Table 2:** Trial structure of the study

Activity/Assessment	Pre visit	T0	T1	T2
	During hospitalization	Visit 1	Visit 2	Visit 3
		Baseline (before discharge)	After 4 months	After 2 further months
Prescreening consent	X			
Consent Form		X		
Inclusion/Exclusion Form	X			
Arterial blood gases		X	X	
Body Mass Index (BMI)		X	X	
6MWT		X	X	X
MRC scale		X	X	X
Borg scale		X	X	X
Barthel Index		X	X	X
PASE		X	X	X
MLHFQ		X	X	X
CAT		X	X	X
Triage to assess the patient’s compliance to the rehabilitation program (only in the intervention group)			X	
Customer satisfaction (only in the intervention group)			X

1. Evaluation of exercise capacity by the 6-min Walk Test (6MWT) [22]
2. Assessment of dyspnea in activities of daily living (ADL) by the Medical Research Council (MRC) scale [23]
3. Assessment of dyspnea and muscle fatigue in ADLs by the Borg scale [24] – patients were asked to report their own sensations referring to the previous day
4. Evaluation of disability by the Barthel Index [25]
5. Indirect assessment of physical activity by the Physical Activity Scale for the Elderly (PASE) [26]
6. Assessment of QoL by the Minnesota Living with Heart Failure Questionnaire (MLHFQ) [27] and by the COPD Assessment Test (CAT) [28]

Then, patients were randomized into one of the two groups. The same measurements were repeated after 4 months (T1) and after a further 2 months of follow-up (T2).

Only in the patients of the intervention group, we evaluated:

1. The percentage of the prescribed training sessions that were actually performed (number of performed sessions as a percentage of the total scheduled training sessions); in addition, the PT filled out a 5-point Likert scale (see Table 3) at the T1 visit, assessing the collaboration and compliance of patients in the intervention group

**Table 3:** Triage to assess a patient’s compliance to the rehabilitation program

0	*Nothing*: the patient has never followed the changes to the program of retraining provided by the physiotherapist
1	*Little*: the patient has followed for less than half of the time the information provided by the physiotherapist
2	*Medium*: the patient has followed for about half of the time the information provided by the physiotherapist
3	*Sufficient*: the patient has followed for more than half of the time the information provided by the physiotherapist
4	*Very much*: the patient has always followed the changes to the program of retraining provided by the physiotherapist
2. Satisfaction regarding the assistance was measured by the patient at T1. The six items, with a score from 0 (not at all satisfied) to 4 (very satisfied), enquired about the service as a whole, the use of the devices, the health care professionals’ willingness to respond to the patient’s needs, clarity of the indications and suggestions made by the NT and PT, the feeling of support, and if the service was felt to be of real help or not (Table 4)

**Table 4:** Customer satisfaction

1. How do you judge the system overall?	
a. Not satisfying at all	0
b. Poorly satisfying	1
c. Fairly satisfying	2
d. Quite satisfying	3
e. Very satisfying	4
2. Was it easy to use the devices?	
a. Very complicated	0
b. Quite complicated	1
c. Complicated	2
d. Quite easy	3
e. Very easy	4
3. Did you experience difficulties in contacting the service?	
a. Very frequently	0
b. Frequently	1
c. Sometimes	2
d. Rarely	3
e. Never	4
4. Were the indications of the health staff clear?	
a. Not at all	0
b. Poorly clear	1
c. Fairly clear	2
d. Quite clear	3
e. Very clear	4
5. Do you feel more secure since when you have access to the service?	
a. Not at all	0
b. Poorly	1
c. Fairly	2
d. Much	3
e. Very much	4
6. Did the access to the service help your family or the people you live with?	
a. Not at all	0
b. Poorly	1
c. Fairly	2
d. Much	3
e. Very much	4

### Interventions

#### Intervention group

All patients randomized to the intervention group received an educational intervention from the NT and PT and were followed by both during the home program.

##### Nurse program

Before discharge from the hospital, all patients were given instruction about their disease condition. The home-based telesurveillance program [16, 17] is designed to provide multidisciplinary care through structured telephone support and telemonitoring. An intensive NT care management program was created, and a NT followed the enrolled patients for 4 months. NTs perform a weekly intervention mainly through structured appointments (from Monday to Friday from 8:30 a.m. to 4:00 p.m.), collecting information about disease status (blood pressure and body weight measurements) and symptoms (general information). Patients were provided with a pulse oximeter (GIMA, Gessate (MI), Italy), and a portable one-lead electrocardiograph (Card Guard 2206; Card Guard Scientific Survival Ltd., Rehovot, Israel) and instructed on how to transmit the electrocardiogram (EKG) trace via a fixed or mobile telephone line to the NT. Participants in the study were also informed that additional telesupport from the NT, aside from the normal weekly NT call, could be accessed by the patients themselves in the case of urgent need or emergency. Unscheduled contacts could be made, 24 h/day 365 days/year, to the NT on duty in case of symptoms manifesting, signs of possible decompensation, or for any doubt about therapy. The EKG was transferred during each call to the telemedicine center, which stored it and sent it to the hospital unit. At the end of each telephone call, the NT could provide support to the patient, offering advice regarding diet, lifestyle, and medications, and suggesting changes in therapy within patient-adapted criteria previously defined with the cardiologist or pulmonologist supervising the program, and could fix an appointment for consultation with the specialist and schedule the next telephone appointment. All conversations were recorded; EKG tracings, the patient’s clinical data and any suggestions made were recorded in a personal health electronic record by the NT. Medical doctors were consulted before referring any patient to the Emergency Department.

##### Physiotherapist program

Patients were assessed by the PT who designed a personalized rehabilitation program to meet each patient’s particular needs. The level and intensity of physical activity were calculated in a rehabilitation triage schedule (Table 5) adding together dyspnea, meters walked (6MWT), and ADL scores. Based on this score, patients with a score from 0 to 6 were prescribed hard training, while patients with a score from 7 to 12 were prescribed light training. An educational session was held where the PT instructed the patients and their caregivers on how to perform the exercises correctly and described the rehabilitation goals within the training period. Patients were provided with the required instruments (weights, mini-ergometer or cycle-ergometer, pedometer, and physical activity diary) and were instructed in how to utilize them.

**Table 5:** Rehabilitation triage schedule

Scores	Dyspnea	6MWT	ADL
0	No dyspnea at usual activity, performed at normal speed, or dyspnea only at extraordinary activity		
1x	Dyspnea at harder activity without pauses (climbing > 3 flights of stairs)	>350 m	Independent
2x	Dyspnea at moderate activity with occasional pauses (walking slightly uphill and climbing <3 flights of stairs	350–300 m	Minimal assistance needed
3x	Dyspnea at mild activity and light exercise with many pauses (walking, washing, standing up)	300–250 m	Moderate assistance needed
4x	Dyspnea at rest, sitting, or lying down	<250 m	Totally dependent

6MWT 6-min Walk Test, ADL activity of daily living

In addition, patients were provided with a pamphlet in which their personalized rehabilitation program was recorded, and a DVD, recorded at our institute, which presented a simulation by the PT of all the exercises prescribed. Recorded on the DVD, each patient could find the training path that they followed during their stay in hospital: callisthenic exercises for different body regions (neck, upper and lower limbs, shoulders, torso), exercises for strengthening of the limbs, gluteal and abdominal muscles, stretching and running-on-the-spot exercises and arm-crank exercises for the upper limbs.

##### Rehabilitation program

The rehabilitation program started after in-hospital rehabilitation discharge; in general, this was 7 days after the patient’s enrollment. The number and intensity of training sessions were adapted during the period of the study, or in the case of problems reported by patients, during telephone contacts.

The “light training” rehabilitation program consisted of:

- 15–25 min of exercise with a mini-ergometer without load three times/week
- 30 min of callisthenic exercises three times/week
- Free walking twice a week

The “hard training” rehabilitation program consisted of:

- 30–45 min of mini-ergometer with incremental load (from 0 to 60 watts max), from 3 to 7 days per week
- 30–40 min of muscle reinforcement exercises using 0.5-kg weights, 3 to 7 days per week
- Pedometer-based walking, from 2 to 7 days per week. The pedometer provided numerical feedback so that the number of steps could be increased week by week (increase predicted: 10 % per week)

During the 4 months of training, the program at home was targeted to reach a moderate or high level of dyspnea and/or muscle fatigue according to the Borg scale at the end of any training session. Based on this assessment, the PT could decide to increase or maintain the workload. Patients were asked to write down the physical activity performed daily in a diary and to report it during the next telephone appointment.

The PT contacted the patient by telephone once a week. During each scheduled telephone contact, the PT gathered information about the patient’s general clinical condition (e.g., asthenia, muscle, and joint pain), physical activity performed during the week (duration of exercises and number of steps walked), and clinical parameters before and after training (blood pressure, heart rate, oxygen saturation, and Borg scale score). The PT verified the training level of physical activity performed and planned the rehabilitation targets for the following week (weight use, increase in number of steps, and duration of exercise). During the telephone call, the PT also gave extra reinforcement on the value of lifestyle changes and the importance of exercise. At the end of each call, the PT entered the data into a personal health electronic record.

#### Control group

On discharge from in-hospital rehabilitation, patients in the control group received the standard care program including medications and oxygen prescription, visits from the general practitioner, and in-hospital check-ups on demand. Patients were free to conduct physical activity without any monitoring or reinforcement provided by the hospital. At study enrollment, patients were instructed in an educational session about the desirability of maintaining a healthy lifestyle and were invited to practice daily physical activity as preferred.

### Telemedicine Service Center

A Telemedicine Service Center (HTN, Brescia, Italy) provided technological support, biomedical devices, and a call center to provide telemonitoring activities, a database for the data collected (TelMed platform), and clinical and nursing support during the night and at weekends through its health personnel, thus guaranteeing the 24 h/day service.

### Outcome assessments

The primary outcome was exercise tolerance improvement measured by difference in the meters walked in the 6MWT. The secondary outcomes were: (1) reduction of hospitalizations for cardiovascular and/or respiratory diseases, (2) reduction of hospitalizations for all causes, (3) improvement of QoL in the MLHFQ and the CAT, (4) reduction in impairment/disability evaluated by the Barthel Index, (5) reduction in dyspnea evaluated by the MRC scale, (6) reduction in dyspnea and fatigue at rest evaluated by the Borg scale, (7) improvement of physical activity profile evaluated by the PASE questionnaire and daily steps reported by patients, and (8) improvement of oxygenation (PaO_2_/FiO_2_).

In the intervention group only, we also evaluated: (1) adherence to at least 70 % of the prescribed rehabilitation sessions, (2) qualitative evaluation of patients’ compliance to the rehabilitation program, (3) use of health services, calculated as total and per-person number of PT and NT scheduled and unscheduled calls, total and per-person number of PT home visits, total and per-person number of educational sessions, and total and per-person time spent by the PT and NT in the study.

### Withdrawal

According to Italian research ethics legislation, we informed the patients about their rights as subjects in a scientific trial and about their discontinuation rights. We do this to make patients consider participation thoroughly to diminish the likelihood of their dropping out. Patients can withdraw from the trial at their own request or at the request of their legal representative at any time. Every withdrawal was recorded in the patient’s “personal health record” (TelMed).

### Data collection, management, and analysis

We collected and managed study data using a shared web platform (TelMed) made available by the Telemedicine Service Center. Only relevant staff have logged access to the key file.

The principal investigators from each scientific discipline have a shared responsibility to secure and monitor data collection and interpretation, and thus they are all involved in project management, analysis of samples, data collection, and observations and will all have access to the final dataset and be jointly involved in the interpretation of results.

### Adverse events monitoring

All adverse events that occurred during the 6-month study observation period will be reported in the final paper. A serious adverse event is defined as any untoward medical occurrence resulting in hospitalization or prolongation of hospitalization, or which results in a life-threatening problem, death, or disability. Adverse events will be defined as any untoward occurrences in study participants, potentially related to implementation of the study protocol. All serious and unexpected adverse events will be reported to the Ethics Committee as required.

### Sample size

A priori sample size was calculated based on previously published randomized controlled trial (RCT) data on COPD and CHF rehabilitation [29, 30] using primary outcome. With an estimated improvement at T1 with respect to T0 in the treatment group of 30 ± 50 m (mean ± standard deviation (SD)) on the 6MWT and no change expected in the control group (0 ± 50 m), at 80 % of power and a significance level of *p* < 0.05, our RCT would need a sample size consisting of at least 44 participants in each group. By taking the probability of dropouts into account (20–25 % of enrolled patients), we decided to include at least 55–60 patients in each group.

### Statistical analysis

Statistical analysis will be carried out by a certified health professional using STATA 13.0 software (College Station, TX, USA). Data will be descriptively analyzed and presented as percentage or mean ± standard deviation for all clinical variables, median ± interquartile (IQ) range for variables without a normal distribution, and percentage for categorical and binary variables. Distribution and normality of variables will be tested by the Kolmogorov-Smirnov test. To compare groups at T0, Student’s *t* test or the Mann-Whitney-Wilcoxon test for continuous variables, and the chi-squared test for categorical variables, will be used.

The effects of training will be analyzed by two-way analysis of variance (ANOVA) for repeated measures (time and group). A post hoc analysis will be conducted when the ANOVA F ratio is significant for Student’s *t* test among times and groups, and Bonferroni’s correction will be applied.

Also, predictive analysis will be performed to detect the characteristics of improvers and nonimprovers using multivariate logistic regression.

The level of significance will be set at *p* < 0.05.

## Discussion

With the progressive ageing of the general population, there is a need to develop more intensive and integrated rehabilitation interventions for the growing number of more complicated, disabled, high-risk patients. The aim of our study was to provide patients with combined COPD/CHF (complex and frequently frail patients with a very high risk of rehospitalization) with an integrated, multidisciplinary NT- and PT-oriented program at the time of in-hospital rehabilitation discharge. This would be characterized by improved knowledge of the signs and symptoms of their disease, with interventions on self-management in the use of medications, evaluation and solution of problems related to exacerbations according to a personalized action plan (self-management), continuous education at a distance with the possible use of a specialist network for a second opinion via remote surveillance (self-guided), home visits as needed, and a guided rehabilitation maintenance program (home care).

Despite the increasing incidence of chronic diseases and, among them, the high prevalence of CHF and COPD, there are no studies in the literature dealing with a similar home-care approach supported by telemedicine in this patient population, where the coexistence of both diseases implies a far greater functional deficit and impact on QoL than in other chronic conditions. Through this program, the NT and PT, with the support of the physician, actively monitored the clinical situation and the patient’s physical status, modifying the therapeutic approach and the workload weekly.

To minimize bias we used consecutive enrollment and central randomization. Due to the nature of the trial it was not possible to blind patients and health care personnel. We tried to standardize as much as possible the nursing and physiotherapy approach in the three hospitals involved in patient enrollment, conducting joint training of staff, organizational meetings and planning before commencing patient enrollment. The rehabilitation program adopted at home tried to reproduce as much as possible the rehabilitation carried out in the hospital, at the same time personalizing the program to the needs of patients.

The choice of the 6MWT as a primary outcome measure was motivated by the fact that this is a very sensitive test to treatments offered in both these diseases and is among the most frequently used in the literature. The test measures the distance covered when subjects are instructed to walk as quickly as they can for 6 min, and performance on this test has been used as a measure of cardiovascular exercise capacity, particularly in patients with congestive heart failure, and chronic lung disease. The distance covered during the 6MWT has been shown to be a sensitive measure of heart disease severity and is a useful predictor of mortality [31].

We also considered it important to monitor dyspnea and muscle fatigue [23, 32] using the Borg scale; even if this is a subjective index, it gives important information on the health status of the patient at any time of the study. We chose the PASE [26] as a tool to measure the level of physical activity, as our population is aged 65 years and older. The PASE consists of self-reported occupational, household, and leisure items over a 1-week period and can be administered by telephone, mail, or in person. The PASE scoring was derived from movement counts from an electronic physical activity monitor, activity diaries, and self-assessed activity levels in a general population of noninstitutionalized older persons. The PASE can be used to measure physical activity in surveys of older people and to assess the effectiveness of interventions.

The evaluation of QoL is an important indicator of the effect of the home program and it remains poorly investigated in telehealth. Most published trials have been powered to highlight the effectiveness in “hard” endpoints such as mortality and cardiovascular or respiratory disease-related hospitalizations; consequently, information on the efficacy of such programs on surrogate outcomes, physical performance, and QoL are lacking [33, 34]. Other major indexes include patient satisfaction [35] and adherence to the program carried out [36–38], which we included in our study.

This is a real-life study, in which the complexity, frailty, age, high risk of exacerbations, and high variability in the individual response of the patients enrolled in the home program can have important effects on the expected results.

### Trial status

Enrollment of patients started in July 2013 and ended in October 2014. Follow-up ended in April 2015. The data, currently being processed, will show whether telehealth management in combined COPD/CHF – complex, frail patients at high risk of exacerbations – is effective.

## Additional file

Additional file 1: SPIRIT checklist. (DOC 122 kb)

## Abbreviations

6MWT: 6-min Walk Test
ADL: Activities of daily living
BMI: Body Mass Index
CAT: COPD Assessment Test
CHF: Chronic heart failure
COPD: Chronic obstructive pulmonary disease
MLHFQ: Minnesota Living with Heart Failure Questionnaire
MRC: Medical Research Council
NT: Nurse-tutor
PASE: Physical Activity Scale for the Elderly
PT: Physiotherapist
QoL: Quality of life
